# Shrimp ponds lead to massive loss of soil carbon and greenhouse gas emissions in northeastern Brazilian mangroves

**DOI:** 10.1002/ece3.4079

**Published:** 2018-05-04

**Authors:** J. Boone Kauffman, Angelo F. Bernardino, Tiago O. Ferreira, Nicholas W. Bolton, Luiz Eduardo de O. Gomes, Gabriel Nuto Nobrega

**Affiliations:** ^1^ Department of Fisheries and Wildlife Oregon State University Corvallis Oregon; ^2^ Department of Oceanography Federal University of Espírito do Santo Vitória ES Brazil; ^3^ Soil Science Department Luiz de Queiroz College of Agriculture University of São Paulo Piracicaba SP Brazil; ^4^ D.B. Warnell School of Forestry and Natural Resources University of Georgia Athens Georgia; ^5^ School of Forest Resources and Environmental Science Michigan Technological University Houghton Michigan

**Keywords:** blue carbon, carbon loss, land use carbon footprint, tidal wetlands, tropical wetlands

## Abstract

Mangroves of the semiarid Caatinga region of northeastern Brazil are being rapidly converted to shrimp pond aquaculture. To determine ecosystem carbon stocks and potential greenhouse gas emissions from this widespread land use, we measured carbon stocks of eight mangrove forests and three shrimp ponds in the Acaraú and Jaguaribe watersheds in Ceará state, Brazil. The shrimp ponds were paired with adjacent intact mangroves to ascertain carbon losses and potential emissions from land conversion. The mean total ecosystem carbon stock of mangroves in this semiarid tropical landscape was 413 ± 94 Mg C/ha. There were highly significant differences in the ecosystem carbon stocks between the two sampled estuaries suggesting caution when extrapolating carbon stock across different estuaries even in the same landscape. Conversion of mangroves to shrimp ponds resulted in losses of 58%–82% of the ecosystem carbon stocks. The mean potential emissions arising from mangrove conversion to shrimp ponds was 1,390 Mg CO_2_e/ha. Carbon losses were largely from soils which accounted for 81% of the total emission. Losses from soils >100 cm in depth accounted for 33% of the total ecosystem carbon loss. Soil carbon losses from shrimp pond conversion are equivalent to about 182 years of soil carbon accumulation. Losses from mangrove conversion are about 10‐fold greater than emissions from conversion of upland tropical dry forest in the Brazilian Caatinga underscoring the potential value for their inclusion in climate change mitigation activities.

## 1 INTRODUCTION

Mangrove forests are coastal ecosystems with a unique biodiversity providing many ecosystem services including functions as important global carbon sinks (Alongi, 2014; Donato et al., 2011; Kristensen et al., 2008; UNEP 2014). Occurring in 118 countries, Giri et al. (2011) reported that globally mangroves cover 137,760 km^2^ of coastal area. Because of the combination of high net ecosystem productivity and low decomposition rates, mangroves frequently sequester large quantities of carbon in soils. Globally, the average carbon stock of mangrove forests is about 885 Mg C/ha (Kauffman & Bhomia, 2017). These results suggest that there is an estimated 10.8 Pg of carbon stored in the extant mangroves of the world.

There are about 1,071,084 ha of mangroves in Brazil (Magris & Barreto, 2010) which is more than any other nation in the Americas and about 7% of the world's total (Giri et al., 2011). Over 80% of the mangroves of Brazil are found along the northern coast from the states of Ceará in the east to Amapá in the west. The concentration of mangroves along the equatorial Brazilian coastline is among the highest on earth. While mangroves are widespread in Brazil, we know of no studies that have reported ecosystem carbon stocks for this region. However, the several studies reporting aboveground or belowground carbon stocks of tropical and subtropical mangroves and salt marshes in Brazil suggest they are important carbon sinks (Ferreira et al., 2010; Sanders, Smoak, Naidu, & Patchineelam, 2008; Sanders, Smoak, Naidu, Sanders, & Patchineelam, 2010; Sanders, Smoak, Naidu, Araripe et al., 2010; Suárez‐Abelenda et al., 2014; Santos et al., 2017).

Despite the importance of mangroves as carbon sinks and the ecological services they provide (Costanza et al., 2014; UNEP, 2014), they are vulnerable to loss through coastal development, pollution, and climate change (Pendleton et al., 2012; Servino, Gomes, & Bernardino, 2018). Brazil is no exception. Nearly 50,000 ha of mangroves in Brazil have been converted to other land uses (4% of total mangrove area; e.g., Bernardino, Gomes, Hadlich, Andrades, & Correa, 2018) with shrimp farming responsible for 20%–50% of the total converted area (FAO, 2007; Lacerda, 2006). Degradation of coastal ecosystems by this land use is not limited to the confines of the shrimp ponds. Mangroves near shrimp ponds are also greatly impacted by effluents that result in changes in soil biogeochemistry such as enrichment of N and P and increased greenhouse gas (GHG) emissions from soils (Nóbrega, Ferreira, Romero, Marques, & Otero, 2013; Nóbrega et al., 2016; Suárez‐Abelenda et al., 2014).

While >80% of the shrimp ponds in Brazil are found in the northeastern part of the country, little data exist on the influences of conversion to aquaculture on carbon losses or greenhouse gas emissions (Lacerda, 2006). To better understand the potential values of these ecosystems in climate change mitigation strategies and to document the influences of current land uses as a source of GHG emissions, the objectives of this study were to quantify carbon stocks of mangroves vulnerable to conversion to shrimp ponds, determine carbon losses by conversion, and estimate potential cumulative carbon emissions from this conversion.

## 2 METHODS

### 2.1 Study site

The study was located in mangroves of the semiarid region of northeastern Brazil. The uplands of the region are dominated by deciduous tropical dry forests that are densely populated by people living at or below the subsistence level. Tropical dry forests such as this landscape comprise ≈42% of all areas occupied by tropical or subtropical forests (Murphy & Lugo, 1986).

**Figure 1:**
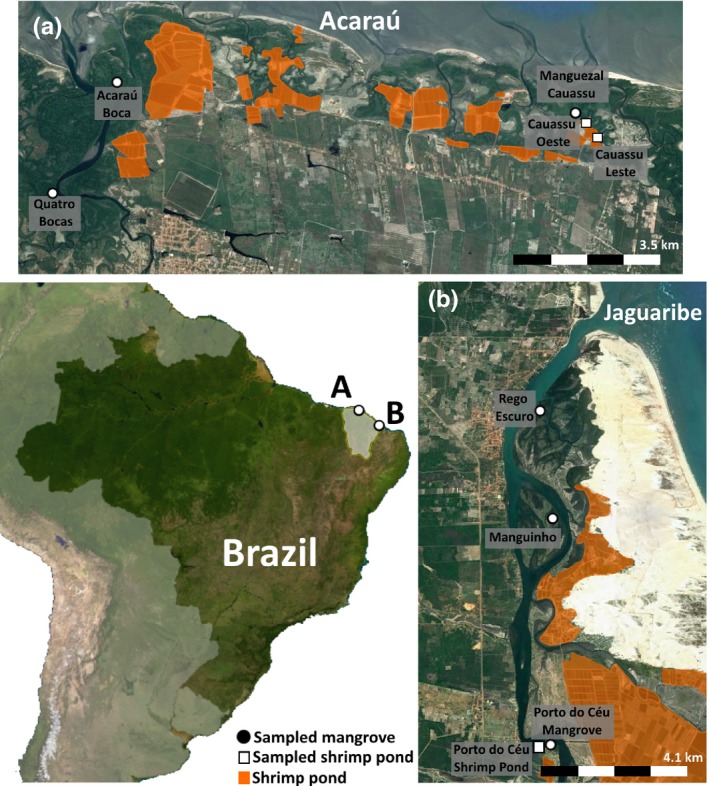
Study sites and sample locations in Ceará State, Brazil. “A” is the Acaraú Estuary and “B” is the Jaguaribe Estuary

The study areas were located in estuaries of the Jaguaribe and Acaraú rivers in the state of Ceará. The development of the shrimp industry in this region has supplanted many mangroves and other coastal ecosystems (Figure 1). The mean annual temperature at the mouth of the Rio Jaguaribe is 27.1°C and the rainfall averages 1,024 mm. In Acaraú, the average annual temperature is 27.7°C and rainfall averages 1,203 mm (Alvares, Stape, Sentelhas, Gonçalves, & Sparovek, 2013; Bernardino et al., 2015).

In each of these estuaries, we sampled three intact mangroves that were located in the upper, mid, and lower portion of each of the estuaries (Figure 1). In addition to mangroves, we sampled three shrimp ponds that had been formed from and were surrounded by mangroves. These ponds were paired with sampled mangroves that were adjacent to the ponds. Based upon interviews with local people, the Cauassú Leste and Cauassú Oeste ponds that we sampled in the Acaraú Estuary had been established about 10–12 years before sampling and were still active. The sampled Porto Céu pond in the Jaguaribe Estuary had been formed, but then abandoned 8 years prior to sampling. The Cauassú Leste and Cauassú Oeste were paired with the adjacent Manguezal Cauassú site and the Porto Céu site was paired with the Porto Céu mangrove. As the ponds were immediately adjacent to the mangroves and occurring on geomorphically similar surfaces, we assumed the differences in carbon stocks would reflect the losses due to land conversion.

### 2.2 Field sampling

All sampled mangroves were estuarine (or riverine following the geomorphic classification of Lugo & Snedaker, 1974) with canopy heights exceeding 10 m (Table 1). Within each site, ecosystem carbon stocks (aboveground and belowground) were measured following methodologies outlined by Kauffman and Donato (2012). At each mangrove and shrimp pond site, six plots were established 20 m apart along a 100 m transect positioned in a perpendicular direction from the mangrove/estuary ecotone. At each plot, we collected data necessary to calculate total carbon stocks derived from standing tree biomass, downed wood (dead wood on forest floor), and soils to the depth of an indurated horizon composed of marine sands.

**Table 1:** Sites sampled in Ceará Brazil January 2016. Soil depth, salinity, pH, tree density, and basal area are reported as mean ± one standard error

Site name	Cover type	Dominance	Latitude	Longitude	Soil depth (cm)	Salinity (‰)	pH	Tree density (ha)	Basal area (m^2^/ha)
Rio Jaguaribe									
Rego Escuro	Tall mangrove—lower end of estuary	Laguncularia‐ Rhizophora	−04°28.623′	−37°46.769′	156 ± 11	49 ± 2	6.8 ± 0.1	2,187 ± 391	20.2 ± 2.1
Manginho	Tall mangrove—midestuary	Rhizophora‐Avicennia	−04°26.933′	−37°47.177′	169 ± 16	28 ± 3	nd	5,714 ± 4,006	22.2 ± 2.7
Porto Céu Mangrove	Tall mangrove—upper estuary	Laguncularia	−04°32.301′	−37°46.864′	61 ± 6	nd	6.3 ± 0.2	6,650 ± 945	34.5 ± 9.9
Porto Céu Shrimp Pond	Abandoned shrimp pond	Laguncularia seedlings	−04°32.396′	−37°46.985′	60 ± 9	nd	nd	20,557 ± 18,419	1.4 ± 1.2
Rio Acaraú’									
Quatro Bocas	Tall mangrove—midestuary	Rhizophora	−02°52.114′	−40°08.714′	210 ± 6	40 ± 1	nd	1,602 ± 443	21.1 ± 2.8
Acaraú Boca	Tall mangrove—lower end of estuary	Rhizophora‐ Avicennia	−02°50.629′	−40°08.024′	255 ± 13	41 ± 0	6.8 ± 0.1	1,015 ± 407	18.6 ± 2.7
Manguezal Cauassú	Tall mangrove—lower end of estuary	Rhizophora‐Avicennia	−02°50.999′	−40°01.884′	239 ± 7	45 ± 1	6.2 ± 0.2	1,397 ± 411	18.5 ± 1.5
Cauassú Oeste	Active shrimp pond	Bare ground	−02°51.078′	−040°01.753′	103 ± 15	46 ± 6	7.1 ± 0.1	0 ± 0	0 ± 0
Cauassú Leste	Active shrimp pond	Bare ground	−02°51.268′	−040°01.628′	144 ± 45	53 ± 5	6.5 ± 0.3	0 ± 0	0 ± 0

#### 2.2.1 Biomass of trees and shrubs

Four species of mangroves were encountered in the sampled mangrove stands: *Rhizophora mangle* L. (Rhizophoraceae), *Avicennia germinans* (L.) Stearn (Avicenniaceae), *Laguncularia racemosa* (L.) Gaertn., (Combretaceae) and *Avicennia schaueriana* (L.) Stearn (Avicenniaceae). Composition, tree density, and basal area of the mangroves were quantified through identification of the species and measurements of diameter at 1.3 m height (diameter at breast height, hereafter dbh) of all trees rooted within each plot of each transect. Plot size for tree measurements was 154 m^2^ (7 m radius) for trees >5 cm dbh and a nested plot with a radius of 2 m for trees with a dbh of <5 cm. The diameter of trees of *R. mangle* was measured at the main branch, 30 cm above the highest prop root.

Allometric equations were used to calculate tree biomass based on several equations specifically developed for the species encountered in this study. Ideally, the allometric equations utilized should be species‐specific, encompass the range in tree diameters of the study, and come from similar environmental conditions. For *L. racemosa,* we used an equation developed in Florida by Smith and Whelan (2006). For *R. mangle* and *A. germinans,* we used the equations developed in French Guiana by Fromard et al. (1998). These equations were selected for analysis as they represented the best combination of diameter range and sample size. While species‐specific equations encompassing the range in diameter of the trees encountered in this study would likely yield most accurate estimates, variation in tree structure related to environmental conditions may introduce uncertainly in estimates of tree mass especially for larger diameter trees (Kauffman & Donato, 2012). The trees used to develop the allometric equations in the Fromard et al. (1998) study were from a region of South America receiving a greater amount of precipitation than our study. To test for potential differences due to allometric equations, we also analyzed aboveground carbon stocks using allometric equations for mangroves from the state of Pernambuco, Brazil (Medeiros & Sampaio, 2008). This site has similar climatic conditions to our study sites but equations only covered stem diameters <21 cm.

Belowground root biomass for mangrove trees was calculated using the formula developed by Komiyama, Poungparn, and Kato (2005). Tree carbon content (C) was calculated by multiplying biomass by 0.48 for aboveground and 0.39 for belowground biomass (i.e., the mean carbon concentration of mangrove plant tissues; Kauffman & Donato, 2012). Standing dead trees were included in aboveground biomass calculations. For each dead tree, the dbh was measured and assigned to one of three decay classes: Status 1—dead trees without leaves, Status 2—dead trees without secondary branches, and Status 3—dead trees without primary or secondary branches (Kauffman & Donato, 2012). Biomass of class I dead trees was estimated to be 97.5% of a live tree, class II—80% of a live tree, and class III—50% of a live tree.

#### 2.2.2 Downed wood

We used the planar intersect technique adapted for mangroves to calculate mass of dead and downed wood (Adame et al., 2013; Kauffman & Donato, 2012). At the center of each plot, four 14‐m transects were established. The first was established in a direction that was offset 45° from the azimuth of the main transect. The other three were established 90° clockwise from the first transect. Along each transect, the diameter of any downed wood intersecting the transect was measured. Downed wood ≥2.5 cm but <7.5 cm in diameter at the point of intersection was measured along the last 5 m of the transect. Downed wood ≥7.5 cm in diameter at the point of intersection was measured from the second meter to the end of the transect (12 m length in total). Large downed wood was separated in two decay categories: sound and rotten. Wood was considered rotten if it visually appeared decomposed and broke apart when impacted. To determine wood mass, we used data of specific gravity of downed wood from mangroves of the Yucatan, Mexico, and reported by Adame et al. (2013). Downed wood was converted to C using factor of 0.50 (Kauffman & Donato, 2012).

#### 2.2.3 Soil carbon

At each plot, fixed‐volume soil samples were collected for bulk density and nutrient concentration using a peat auger consisting of an open‐faced cylindrical chamber with a 6.4 cm radius. This auger is efficient for collecting relatively undisturbed cores from wet soils in mangroves (Donato et al., 2011). The core was systematically divided into depth intervals of 0–15 cm, 15–30 cm, 30–50 cm, 50–100 cm, and >100 cm (if parent materials or an indurated horizon were not encountered before 100 cm depth). At each sampling site, the depth to an indurated horizon was measured. The soil depth was measured at three locations near the center of each plot using a graduated aluminum probe. When soils were >3 m in depth, we limited the calculation of soil carbon pools to 3 m. Samples of a known volume were collected in the field, dried at 60°C to constant mass, and then weighed to determine bulk density. Laboratory analysis was conducted at the University of Sao Paulo and at the Seagrass Analytical Lab, Florida International University, Miami, USA. Soil concentration was determined using a Thermo Flash EA 1112 series C‐N Soil Analyzer. A total of 234 soil samples were collected in this study and analyzed for total carbon. We took 25 random soil subsamples to determine the contribution of the inorganic fraction of carbon to the total. The inorganic carbon fraction was determined using methods outlined in Fourqurean et al. (2012). From these samples, we found that inorganic carbon comprised a mean of 5.7 ± 2.2% of the total soil carbon. Therefore, the organic soil carbon mass was determined by multiplying the total soil carbon concentration by 0.943. Bulk density and organic carbon concentration were then combined with plot‐specific soil depth measurements to determine the soil organic C stocks.

We sampled interstitial salinity and pH of the ground water collected in the bore holes using methods described in Kauffman and Bhomia (2017). A portable handheld refractometer (VEE GEE STX‐3, range—0 to 100 parts per thousand) and pH meter (Milwaukee Instruments, Inc., pH56, pH–Temperature meter) were used for measuring salinity and pH of the soil pore water. Care was taken to ensure that no surface water mixed with the sampled soil porewater as surface water was usually lower in salinity. Porewater was sampled at each soil sampling plot (*n* = 6 in each sampled stand).

### 2.3 Emissions from conversion of mangroves to shrimp ponds

We calculated the potential emissions from conversion of mangrove as the difference between the carbon stocks of mangrove and paired shrimp ponds. The Intergovernmental Panel on Climate Change (IPCC) protocol for tracking changes in carbon stocks and predicting emissions from land cover change in forestry includes the stock‐change approach (IPCC 2003). Using this approach, we calculated cumulative potential emissions that had occurred from the time of mangrove deforestation until the time of sampling. Included in this analysis were losses from all aboveground biomass and the entire soil profile (or a default depth of 3 m when soils exceeded this depth).

Differences in carbon stocks were converted to emissions using the formula:

$$\Delta \text{CLU}=\Delta C_{\mathrm{AB}}+\Delta C_{\mathrm{BB}}+\Delta C_{\mathrm{DW}}+\Delta C_{\mathrm{SOC}}$$

where: CLU = carbon stocks (or total carbon emissions or sequestration) due to land use; C_AB_ = aboveground biomass carbon pool; C_BB_ = belowground biomass carbon pool; C_DW_ = dead wood carbon pool; C_SOC_ = soil organic carbon partitioned into sampled soil depths.

The ecosystem losses are reported as potential CO_2_ emissions, or CO_2_ equivalents (CO_2_e)—obtained by multiplying C values by 3.67, the molecular ratio of CO_2_ to C. While reported as the CO_2_e, these estimates account only for changes in ecosystem C in situ. While likely to be small compared to greenhouse gas emissions, some of the carbon lost in the shrimp ponds conversion may be transferred to other communities via erosion, groundwater transfer, or surface water transfer when ponds are drained for shrimp harvest.

Differences between carbon stocks in mangroves and emissions from shrimp ponds were tested with analysis of variance (ANOVA). If the ANOVA was significant, a least significant differences test was performed to determine which means were significantly different.

## 3 RESULTS

**Figure 2:**
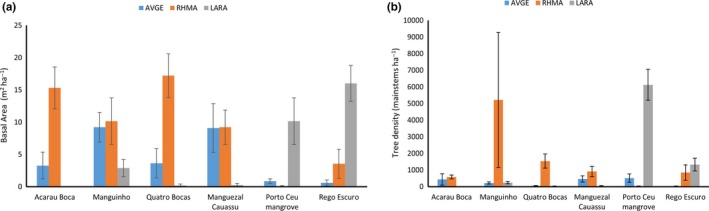
(a) The basal area (m^2^/ha) and (b) density (main stems/ha) of mangroves sampled in Ceará, Brazil (AVGE = *Avicennia germinans*, RHMA = *Rhizophora mangle*, and LARA = *Laguncularia racemosa*). Vertical bars represent ± one standard error

While mangroves in this region are only dominated by only a few species, there was structural variability among and within the sites (Figure 2). The Cauassú and Maguinho sites were co‐dominated by *R. mangle* and *A. germinans*. The Acaraú Boca and Quatro Bocas sites were largely dominated by *R. mangle*. The Porto Céu and Rego Escuro sites were dominated by *L. racemosa*. Density exceeded 5,000 trees/ha in the Manguinho and Porto Céu sites which were located at the upper reaches of the estuaries. In contrast, density of the Acaraú Boca site located in the lower end of the estuary was <1,000 ha^−1^ (Figure 2, Table 1). The active shrimp ponds were devoid of vegetation, but the Porto Céu abandoned shrimp pond had some dense patches of mangrove seedlings with a mean density of 20,557 ha^−1^.

Within stand variation (variation among the different plots) in composition and structure is apparent by the high error terms of the sites (Figure 2). The basal area of the mangroves ranged from 11.1 m^2^/ha at the Porto Céu site to 22.2 m^2^/ha at Manguinho. The very small size of the trees in the Porto Céu abandoned shrimp pond site resulted in a basal area of 1.4 m^2^/ha (Table 1).

The carbon sequestered in mangrove trees ranged from 53 to 114 Mg C/ha (Table S1). The mean aboveground tree carbon in mangroves was 70 Mg C/ha. Downed wood was a minor component of mangroves ranging from 2.3 to 7.7 Mg C/ha.

Soil total carbon concentration in surface soils ranged from 0.8% to 6.5% in the mangroves. Soil carbon concentrations were relatively stable with depth among the sites (Table 2). The notable exceptions were sites in the Acaraú Estuary such as Acaraú Boca, Quatro Bocas, and Cauassú Leste Shrimp pond which had buried historic surface horizons. In these cases, we found some increases in carbon concentrations at depths >100 cm. Soil organic carbon pools of the sampled mangroves ranged by over 10‐fold from 53 Mg C/ha in the Porto Céu mangrove to >600 Mg C/ha in the Manguezal Cauassú site. This is largely reflective of the shallow soils of the Porto Céu sites (about 60 cm) compared to mean depth of 239 cm at Cauassú mangrove and 255 cm at Acaraú Boca (Table 1). We did observe some differences between the estuaries with the three mangroves in the Acaraú estuary having soil carbon pools exceeding 338 Mg C/ha while the three sampled mangroves in the Jaguaribe watershed had total soil carbon pools ≤200 Mg C/ha; Table 2). Again, this is reflective of differences in soil depth; soils ranged in depth from 60 to 169 cm in the Jaguaribe Estuary and from 210 to 255 cm in the Acaraú Estuary.

**Table 2:** Soil properties of sampled mangroves of Ceará State, Brazil. Data are means and one standard error. Numbers following site names are the mean depth to an indurated horizon (mean depth ± one standard error)

Site/Depth range (cm)	Total carbon (%)		Bulk density (g/cm^3^)		Total carbon density (g/cm^3^)		Organic carbon mass (Mg/ha)	
	Mean	*SE*	Mean	*SE*	Mean	*SE*	Mean	*SE*
Mangroves								
Acaraú Boca/255±13cm								
0–15	1.36	0.17	1.08	0.06	0.015	0.002	20.7	2.9
15–30	1.17	0.15	1.03	0.09	0.012	0.002	16.5	2.4
30–50	1.10	0.16	1.03	0.09	0.011	0.002	20.7	3.2
50–100	1.51	0.22	1.00	0.04	0.015	0.003	72.7	13.9
>100	2.47	0.25	1.21	0.09	0.029	0.003	438.2	60.9
Manguezal Cauassú/239±7cm								
0–15	6.52	0.78	0.44	0.03	0.027	0.002	38.6	2.5
15–30	4.63	0.65	0.55	0.05	0.024	0.002	33.9	2.4
30–50	3.68	0.61	0.60	0.05	0.021	0.001	38.8	2.7
50–100	3.08	0.46	0.74	0.07	0.022	0.004	104.8	17.1
>100	2.49	0.09	1.20	0.05	0.030	0.001	387.4	14.0
Manguinho/169±16cm								
0–15	2.09	0.32	0.83	0.06	0.016	0.001	23.3	1.9
15–30	2.11	0.25	0.82	0.07	0.017	0.001	23.5	1.5
30–50	2.09	0.34	0.76	0.07	0.015	0.002	28.0	2.9
50–100	1.75	0.18	0.86	0.04	0.015	0.002	70.8	7.1
>100	0.88	0.28	1.18	0.14	0.008	0.002	54.8	14.2
Porto Céu Mangrove/61±6cm								
0–15	0.90	0.19	1.34	0.08	0.012	0.002	16.4	2.9
15–30	0.85	0.15	1.24	0.07	0.010	0.001	14.2	1.6
30–50	0.51	0.17	1.36	0.12	0.006	0.001	11.2	2.4
50–100	0.47	0.11	1.50	0.01	0.007	0.002	11.0	7.7
>100	–	–	–	–	–	–	0.0	0.0
Quatro Bocas/210±6cm								
0–15	1.34	0.13	0.99	0.05	0.013	0.001	18.5	1.3
15–30	0.98	0.30	1.02	0.06	0.009	0.002	13.0	2.7
30–50	0.91	0.16	1.03	0.05	0.009	0.001	17.1	2.7
50–100	1.57	0.38	1.02	0.06	0.016	0.004	75.0	18.8
>100	1.64	0.15	1.24	0.05	0.013	0.002	214.9	28.7
Rego Escuro/156±11cm								
0–15	1.86	0.14	0.82	0.04	0.014	0.001	20.2	2.0
15–30	1.88	0.11	0.83	0.03	0.016	0.001	22.0	2.0
30–50	1.67	0.13	0.84	0.04	0.014	0.001	26.7	2.4
50–100	1.68	0.09	0.85	0.05	0.014	0.001	65.8	3.4
>100	0.70	0.58	1.20	0.13	0.008	0.004	45.9	12.7
Shrimp ponds								
Porto Céu Shrimp/60±9cm								
0–15	1.43	0.55	0.92	0.11	0.010	0.003	14.7	4.3
15–30	0.17	0.04	1.44	0.07	0.002	0.001	3.2	0.7
30–50	0.26	0.2	1.37	0.1	0.003	0.002	6.5	3.0
50–100	0.04	0.04	1.59	1.59	0.001	0.001	0.5	0.5
>100	–	–	–	–	–	–	0.0	0.0
Cauassú Leste Shrimp/144±45cm								
0–15	0.15	0.04	1.56	0.11	0.002	0.001	3.1	0.8
15–30	0.34	0.10	1.40	0.13	0.004	0.001	6.2	1.8
30–50	1.18	0.0	1.23	0.2	0.011	0.005	13.5	7.5
50–100	0.06	0.3	1.47	0.1	0.001	0.000	2.1	1.1
>100	5.99	0.7	0.63	0.1	0.038	0.003	257.1	117.1
Cauassú Oeste Shrimp/103±15cm								
0–15	0.79	0.44	1.36	0.11	0.009	0.004	12.3	5.6
15–30	0.35	0.26	1.45	0.12	0.004	0.002	5.3	2.4
30–50	0.21	0.14	1.43	0.13	0.002	0.001	4.1	2.0
50–100	0.28	0.21	1.50	0.07	0.004	0.003	15.8	12.1
>100	0.59	0.59	1.55	0.08	0.009	0.009	13.7	13.7

**Figure 3:**
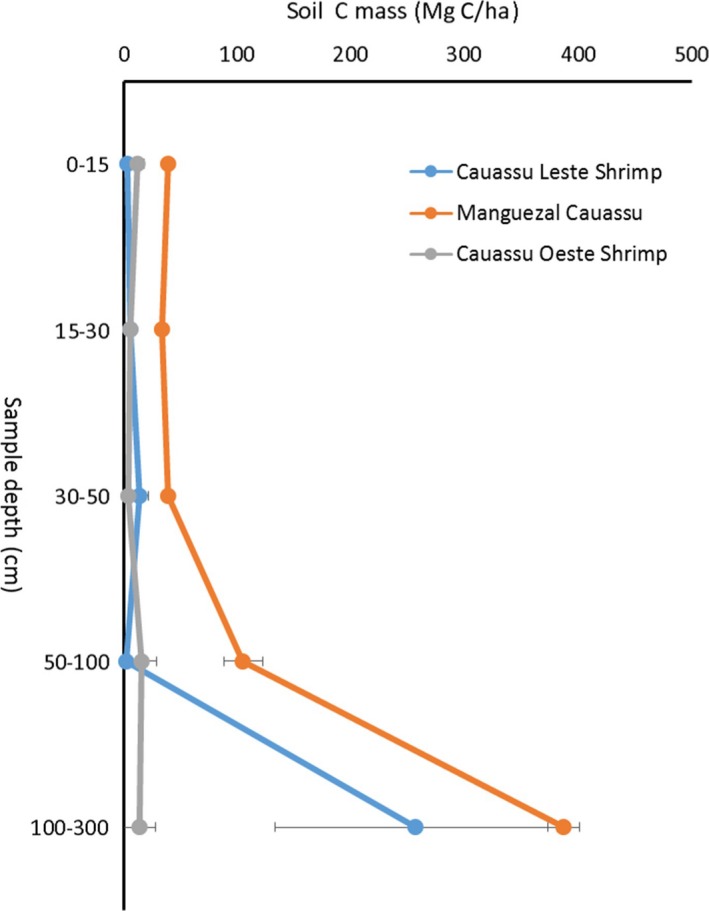
The mass of soils broken down by depth for the mangrove (Manguezal Cauassú) and two adjacent shrimp ponds—Cauassú Leste and Cauassú Oeste. The colored circles represent the mean soil carbon mass for the sampled depths of the entire soil profile. Horizontal bars represent one standard error of the mean carbon mass for the sampled depth

Soil carbon concentrations were lower and bulk densities were higher, when comparing between mangroves and adjacent shrimp ponds (Table 2). For example, the mean soil carbon concentration of all depths combined at the Manguezal Cauassú (mangrove) was 4.1% compared to 0.4% and 1.5% for the adjacent sampled shrimp ponds. The mean soil bulk density was 0.7 g/cm^3^ in this mangrove and >1.26 g/cm^3^ in the nearest shrimp ponds. As a result, there were highly significant differences (*p* = .001) between soil carbon pools in mangroves and adjacent shrimp ponds (Figure 3). The total soil carbon mass in the Cauassú mangrove was 640 Mg C/ha compared to the adjacent shrimp ponds that had soil carbon pools of 54 and 297 Mg C/ha. Similar declines were found for the mangrove/shrimp pond comparison in the Acaraú Estuary. Here, the soil carbon pool of the Porto Céu mangrove was 49 Mg C/ha compared to 29 Mg C/ha in the adjacent sampled shrimp pond.

### 3.1 Ecosystem carbon stocks

**Figure 4:**
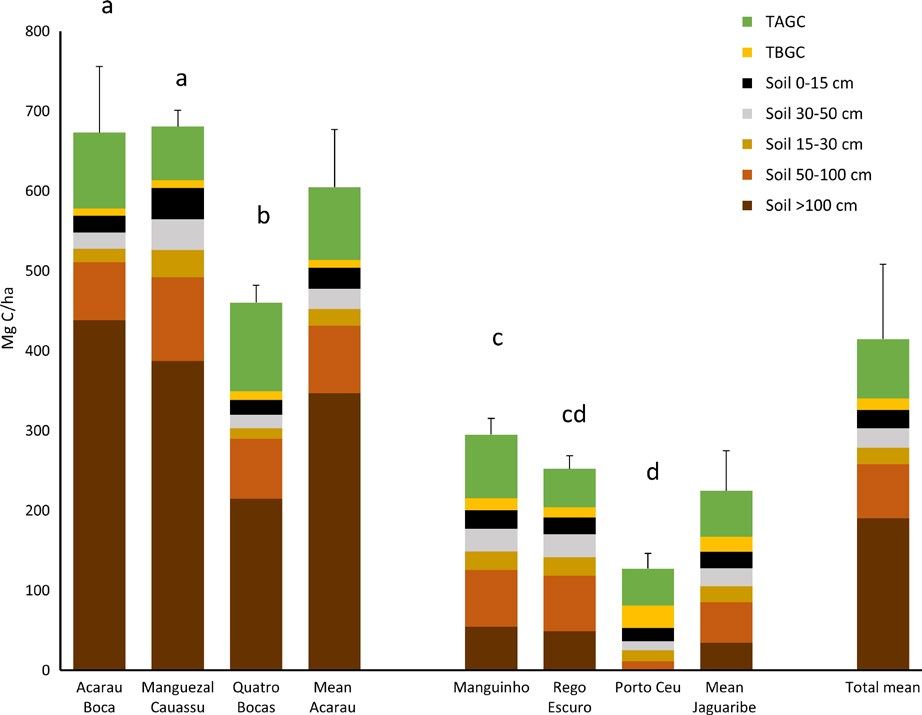
Ecosystem carbon stocks (Mg C/ha) of mangroves sampled in the Rio Acaraú and Jaguaribe estuaries, Ceará, Brazil (TAGC = total above ground carbon pool and TBGC = total below ground plant carbon pool). Vertical bars are one standard error of the mean total ecosystem carbon stock. Different letters above bars signify a significant difference (p < .05) when testing between sites. The means of ecosystem carbon stocks between the Acaraú and Jaguaribe Estuaries were different at p < .0001

The mean ecosystem carbon stock of the northeastern Brazil mangroves was 413 ± 94 Mg C/ha (Figure 4). Soils comprised an average of 81% of the total ecosystem carbon stock. There was a tremendous range in carbon stocks among the mangrove sites varying from 129 at Porto Céu to 681 Mg C/ha at the Cauassú mangrove. Further, we found significant differences in the ecosystem carbon stocks of the mangroves of the Rio Acaraú Estuary (605 Mg C/ha) compared to that of the Rio Jaguaribe Estuary (224 Mg C/ha; *p* = .001). The greatest differences between the two estuaries were in the soil carbon pools >100 cm in depth (347 and 35 Mg C/ha for the Acaraú and Jaguaribe, respectively).

### 3.2 Ecosystem carbon stocks of shrimp ponds

**Figure 5:**
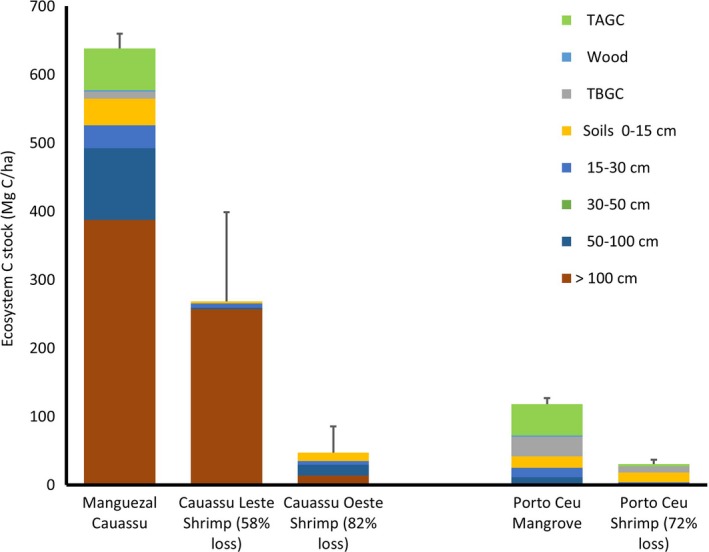
Ecosystem carbon stocks (Mg C/ha) of paired mangroves with adjacent shrimp ponds (TAGC = total above ground carbon pool and TBGC = total below ground plant carbon pool). The Manguezal Cauassú site was paired with two adjacent shrimp ponds (the Cauassú Leste and Oeste shrimp ponds) in the Rio Acaraú Estuary. The Porto Céu mangrove was adjacent to the Port Céu Shrimp pond in the Rio Jaguaribe Estuary. Vertical bars are one standard error of the mean total ecosystem carbon stock

Ecosystem carbon stocks of the shrimp ponds reflect land use and inherent characteristics of the site. Cauassú Leste and Oeste were active shrimp ponds and devoid of aboveground vegetation (Figure 5). The Porto Céu shrimp pond had been abandoned for about 8 years and had a few dense patches of *L. racemosa* seedlings. The ecosystem carbon stock of the Porto Céu shrimp pond was 37 Mg C/ha which represented a 72% loss of the ecosystem carbon stock compared to the adjacent Porto Céu mangrove (Figure 5). In the Acaraú Estuary, the total ecosystem carbon stock of the Manguezal Cauassú mangrove was 681 Mg C/ha. Carbon stocks of the adjacent shrimp ponds were 282 Mg C/ha in the Cauassú Leste and 51 Mg C/ha in the Cauassú Oeste. This represents a decline in ecosystem carbon stocks compared to adjacent mangrove of 58% and 82%, respectively.

### 3.3 Potential carbon emissions from conversion

**Figure 6:**
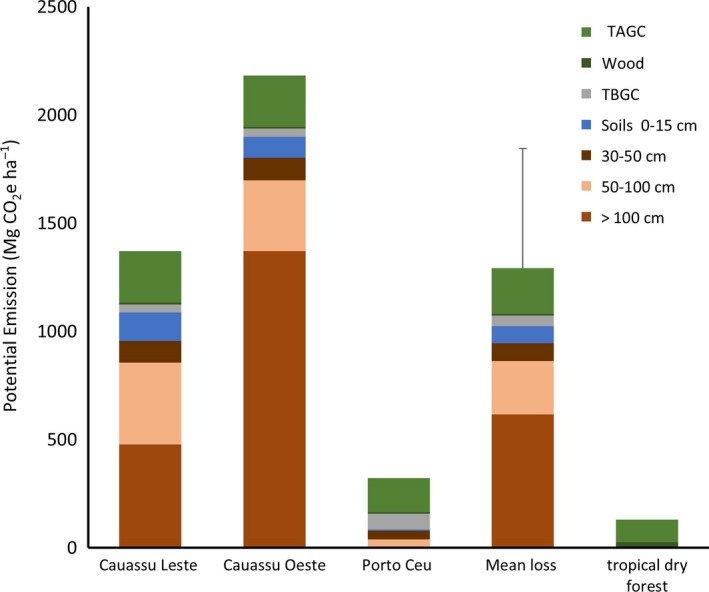
The potential cumulative carbon emissions (Mg CO_2_e/ha) from conversion of mangroves to shrimp ponds, and upland dry forest to shifting cultivation, Ceará, Brazil (TAGC = Total above ground carbon pool and TBGC = total below ground plant carbon pool)

The mean cumulative potential emission from mangrove conversion to the shrimp ponds was 1,371 Mg CO_2_e/ha (Figure 6). This is equivalent to a 72% decline in the total ecosystem carbon stocks of mangroves. However, the range in potential emissions for the sampled ponds was great. Potential emissions from the Porto Céu site that had a low initial carbon stock were 340 Mg CO_2_e/ha. In contrast, potential emissions from the carbon–rich Cauassú Leste site were 2,297 Mg CO_2_e/ha. Declines in carbon stocks occurred not only from the complete loss of aboveground vegetation but in significant losses in soil carbon (Figure 5). Soil carbon losses accounted for 81% of the total emissions. Potential emissions arising from soil carbon at depths exceeding 100 cm were 1,371 Mg CO_2_e/ha at Cauassú Oeste underscoring the importance of sampling at these depths.

## 4 DISCUSSION

The mean ecosystem carbon stocks of the mangroves sampled in this study was 413 Mg C/ha (Figure 3) which is quite low compared to the global mean of mangroves which is 885 Mg C/ha (Kauffman & Bhomia, 2017). Relatively few studies have examined ecosystem carbon stocks of mangroves in estuaries occurring in semiarid or arid landscapes such as those of this study. For example, the mean ecosystem carbon stocks of Yucatan, Mexico mangroves was 663 Mg C/ha in a landscape receiving annual mean rainfall of 1,580 mm (Adame et al., 2013). Similarly, carbon stocks of mangroves of Southern Gabon were 539 Mg C/ha (1818 mm ppt; Kauffman & Bhomia, 2017). In contrast, carbon stocks of the NE Brazil mangroves were lower than those of the arid Solume Delta, Senegal (674 Mg C/ha; 650 mm ppt; Kauffman & Bhomia, 2017), but much greater than ecosystem carbon socks from the hyperarid United Arab Emirates (218 Mg C/ha; ppt <100 mm; Schile et al., 2017). Even though climate, including precipitation, was similar in the Acaraú and Jaguaribe Estuaries, we found significant differences in ecosystem carbon stocks (Figure 4). This underscores the uncertainty of using models based upon precipitation alone to predict ecosystem carbon stocks (Sanders et al., 2017). Further, the uncertainty of using carbon stocks data from one estuary as an estimate of carbon stocks for all estuaries in a region such as is commonly done when scaling estimates to regional or global scales can result in large errors in estimation (Kauffman et al., 2018).

Finally, we found little variation in aboveground biomass estimates using different, but appropriate allometric equations. Using the equations of Fromard et al. (1998) and Smith and Whelan (2006) compared to that of Medeiros and Sampaio (2008) yielded quite similar results. The mean aboveground carbon stocks using the Medeiros and Sampaio (2008) equations was 72 ± 3 Mg C/ha compared to 70 ± 2 Mg C/ha using the Fromard et al. (1998) and Smith and Whelan (2006) equations. The trees accounted for about 19% of the total ecosystem carbon stocks of the mangrove ecosystems of this study and the minor variation in biomass estimates using other allometric equations would not alter our conclusions.

### 4.1 Emissions and losses

In a study of carbon losses and emissions from mangrove conversion to shrimp ponds in four countries, Kauffman et al. (2017) reported the mean potential carbon emission associated with mangrove conversion to shrimp ponds was 1894 Mg CO_2_e/ha which is within the large range of potential emissions sampled in this study (395–2,297 Mg CO_2_e/ha). Similar to results presented here, they also reported that 84% of the greenhouse gas emissions from mangrove conversion came from losses of soil C pools.

We found that with shrimp pond conversion, soil bulk density increased while carbon concentration decreased. This was found throughout the soil profile even at depths >100 cm (Table 2). Soil losses from depths >100 cm in the two Cauassú shrimp ponds were 478 and 1,371 Mg CO_2_e/ha (Figure 6). Similar carbon losses from soils at depths >100 cm were found in Mexican cattle pastures converted from mangroves (Kauffman, Hernandez‐Trejo, Jesus‐Garcia, Heider, & Contreras, 2016) as well as in shrimp ponds in Indonesia (Arifanti, 2017). This underscores the importance of sampling soil carbon at depths exceeding 100 cm in estuaries, where soils are usually much greater than 100 cm to parent materials. In these scenarios, limiting soil sampling to depths ≤100 cm will underestimate the soil carbon susceptible to loss with land conversion, and therefore, also underestimate the greenhouse gas emissions from land use.

The significant losses of carbon from the shrimp ponds reported here are likely underestimates because this land use affects carbon dynamics outside of the boundaries of the shrimp ponds. Suárez‐Abelenda et al. (2014) reported that shrimp pond effluents had dramatic effects on soil carbon storage in affected mangroves surrounding ponds. They found 2.2 times greater carbon stocks in the top 40 cm of soils in mangroves unaffected by wastewater effluents compared to those exposed to such effluents. It is probable that the mangroves sampled adjacent to shrimp ponds in this study are exposed to pond effluents. As such, our estimates may not even reflect the ecosystem carbon stocks of truly undisturbed sites.

The shrimp ponds of this study were intensively managed with high energy use, chemical, and feed inputs that resulted in high productivities of shrimp. Shrimp pond productivity has been reported to be as high as 4,700 kg ha^−1^ year^−1^ in this region (Roubach, Correia, Zaiden, Martino, & Cavalli, 2003). In contrast, productivity for low‐intensity/low‐input extensive shrimp ponds reported by Kauffman et al. (2017) averages about 275 kg ha^−1^ year^−1^. Kauffman et al. (2017) reported that there was an average carbon emission of 1,603 kg of CO_2_e for every kg of shrimp produced from extensive ponds (i.e., the land use carbon footprint). Because of the higher productivity, it could be assumed that the land use carbon footprints arising from the intensive shrimp ponds would be lower. However, the additional greenhouse gas emissions related to the intensive use of electric power for pumping and aeration, emissions from feeds, chemical amendments, antibiotics, and waste water pollution would increase carbon footprints from these operations. For example, Boyd (2005) and Boyd, Tucker, McNevin, Bostick, and Clay (2007) reported that about 1.7 to 2.7 kg of marine fish in the fish meal is required to produce a kg of shrimp. This suggests annual feed inputs equivalent to as much as 12,690 kg of marine fish in the fish meal per hectare of pond to achieve the high productivities of shrimp in this region. Because data are lacking on the effects of shrimp ponds on carbon losses and emissions, we cannot calculate the land use carbon footprint arising from shrimp production in NE Brazil in the same manner as was done for extensive shrimp ponds (Kauffman et al., 2017).

### 4.2 Comparison of emissions and losses with upland tropical dry forest

Carbon pools of the mangrove trees measured in this study greatly exceed that of the upland tropical dry forests surrounding these mangroves. Kauffman, Sanford, Cummings, Salcedo, and Sampaio (1993) reported the aboveground carbon pools of Caatinga forests were about 40 Mg C/ha. In comparison, the mean aboveground carbon pools of the mangroves was 74 Mg C/ha. The large differences in ecosystem carbon stocks are largely below ground where soils in the mangrove were often much deeper than in uplands.

The losses associated with conversion of mangroves to shrimp ponds greatly exceed losses resulting from land cover change in upland tropical forests. For example, carbon losses associated with slash and burn of the upland tropical dry forests of northeastern Brazil were 38.9 Mg C/ha (142.7 Mg CO_2_e/ha; Kauffman et al., 1993; Figure 6). This suggests that the greenhouse gas emissions from conversion of mangroves to shrimp ponds are, on average, almost 10 times greater (range of 2 to 17) than the emissions from upland conversion in northeastern Brazil. This underscores the values of including mangroves in climate change mitigation programs.

Unlike land use in uplands, there a significant loss of soil carbon that has been sequestered in mangrove ecosystems for possibly centuries. Based upon global reviews of soil carbon gain in mangroves, Alongi (2014) calculated the global mean soil carbon accumulation rate for mangroves to be 1.74 Mg C/ha, which is similar to global mean burial rates of 1.34, 2.11, and 1.63 Mg C/ha were calculated by Bouillon, Dehair, Velimirov, Abril, and Borges (2007), Alongi (2009), and Breithaupt, Smoak, Smith, Sanders, and Hoare (2012). In our study, we found that the mean soil loss from the mangroves was 317 Mg C/ha. This suggests that losses due to conversion are equivalent to 182 years accumulation. And, there are additional carbon losses that are occurring outside of the pond perimeter due to influences of effluents on carbon loss (Suárez‐Abelenda et al., 2014). In terms of coastal land use and policy, the ecosystem services of mangroves, including their values as globally important carbon sinks, should be weighed against the short‐term values of production of an export food for developed nations.

## CONFLICT OF INTEREST

None declared.

## AUTHOR CONTRIBUTIONS

BK designed the study, conducted field and laboratory analysis and led the manuscript publication; AFB contributed to study design, field work, and manuscript preparation TF contributed to study design, field work, and manuscript preparation, NB, LEOG, and GNN conducted field work and contributed to manuscript preparation. All agreed to manuscript submission.
