# Supplementary material for: Shrimp ponds lead to massive loss of soil carbon and greenhouse gas emissions in northeastern Brazilian mangroves
Source: Ecol Evol. 2018 May 4;8(11):5530–40. doi: 10.1002/ece3.4079 (PMC6010805; doi:10.1002/ece3.4079)
Supplement: Supplementary file 1 [file ECE3-8-5530-s001.docx]

Supporting Information: Kauffman et al. **Shrimp ponds lead to massive loss of soil carbon and greenhouse gas emissions in Northeastern Brazilian mangroves**

| Supplementary Table 1. Total tree carbon mass, belowground mass and aboveground mass and downed wood of mangroves, Ceará, Brazil. Data are reported as Mg C ha^-1^. | | | | | | | | | | |
| --- | --- | --- | --- | --- | --- | --- | --- | --- | --- | --- |
|  | Total tree carbon | | Belowground | | Aboveground | | Downed wood | |  | |
|  | Mean | SE | Mean | SE | Mean | SE | Mean | SE | |  |
| Acaraú Boca | 98.07 | 17.02 | 9.17 | 1.00 | 88.89 | 16.19 | 5.90 | 2.22 | |  |
| Manguezal Caussau | 75.60 | 5.52 | 10.83 | 1.22 | 64.77 | 5.52 | 2.25 | 0.28 | |  |
| Manguinho | 88.00 | 11.24 | 15.37 | 4.78 | 72.64 | 7.10 | 6.73 | 1.48 | |  |
| Porto Céu mangrove | 73.40 | 6.20 | 27.36 | 2.92 | 46.04 | 3.44 | 2.03 | 0.71 | |  |
| Quatro Bocas | 114.26 | 16.46 | 10.88 | 1.50 | 103.37 | 15.57 | 7.72 | 2.28 | |  |
| Rego Escuro | 54.64 | 8.81 | 12.46 | 0.88 | 42.18 | 8.02 | 6.14 | 2.14 | |  |
| **Mean mangrove** | **83.99** | **2.03** | **14.34** | **0.62** | **69.65** | **2.18** | **5.10** | **1.51** | |  |
|  |  |  |  |  |  |  |  |  | |  |
| Porto Céu shrimp pond | 12.01 | 10.17 | 8.85 | 7.56 | 3.17 | 2.61 | 0.00 | 0.00 | |  |

Supplementary Table 2. Ecosystem carbon stocks (Mg C ha^-1^) of sampled shrimp ponds and mangroves, Ceará Brazil.

|  |  |  |  | |  | | | |  | |
| --- | --- | --- | --- | --- | --- | --- | --- | --- | --- | --- |
| Sites | Aboveground tree | Wood | Belowground tree | Soils 0-15 cm | Soils 15-30 cm | Soils 30-50 cm | Soils 50-100 cm | Soils>100 cm |  | Total ± SE |
| Shrimp Ponds | | | | | | | | | | |
| Cauassú Leste | 0.0 | 0.0 | 0.0 | 3.1 | 6.2 | 13.5 | 2.1 | 257.1 | 282.0 ± 123.0 | |
| Cauassú Oeste | 0.0 | 0.0 | 0.0 | 12.3 | 5.3 | 4.1 | 15.8 | 13.7 | 51.3 ± 36.3 | |
| Porto Céu Shrimp | 3.2 | 0.0 | 8.8 | 14.7 | 3.2 | 6.5 | 0.5 | 0.0 | 36.9 ± 12.9 | |
| Mangrove | | | | | | | | | | |
| Acaraú Boca | 88.9 | 5.9 | 9.2 | 20.7 | 16.5 | 20.7 | 72.9 | 438.2 | 673.0 ± 82.9 | |
| Manguezal Cauassú | 64.8 | 2.3 | 10.2 | 38.6 | 33.9 | 38.8 | 104.8 | 387.4 | 680.8 ± 20.3 | |
| Quatro Bocas | 103.4 | 7.7 | 10.9 | 18.5 | 13.0 | 17.1 | 75.0 | 214.9 | 460. 5± 21.5 | |
| Manguinho | 72.6 | 6.7 | 15.4 | 23.3 | 23.5 | 28.0 | 70.8 | 54.8 | 295.0 ± 20.4 | |
| Rego Escuro | 42.2 | 6.1 | 12.5 | 20.2 | 22.0 | 26.7 | 65.8 | 45.9 | 241.4 ± 16.2 | |
| Porto Céu Mangrove | 46.0 | 2.0 | 27.4 | 16.4 | 14.2 | 11.2 | 11.0 | 0.0 | 129.4 ± 10.0 | |
